# CTL Escape Mediated by Proteasomal Destruction of an HIV-1 Cryptic Epitope

**DOI:** 10.1371/journal.ppat.1002049

**Published:** 2011-05-12

**Authors:** Sylvain Cardinaud, Gesa Consiglieri, Romain Bouziat, Alejandra Urrutia, Stéphanie Graff-Dubois, Slim Fourati, Isabelle Malet, Julien Guergnon, Amélie Guihot, Christine Katlama, Brigitte Autran, Peter van Endert, François A. Lemonnier, Victor Appay, Olivier Schwartz, Peter M. Kloetzel, Arnaud Moris

**Affiliations:** 1 INSERM, UMR-S945, Université Pierre et Marie Curie (UPMC), Paris, France; 2 Institut für Biochemie, Charité-Universitätsmedizin, Berlin, Germany; 3 Institut Pasteur, Unité Cellulaire Antivirale, Paris, France; 4 INSERM, UMR-S943, UPMC, Hôpital Pitié-Salpêtrière, Paris, France; 5 INSERM, U1013, Université Paris Descartes, Faculté de médecine René Descartes, Paris, France; 6 Institut Pasteur, Unité Virus et Immunité, Paris, France; University of Geneva, Switzerland

## Abstract

Cytotoxic CD8+ T cells (CTLs) play a critical role in controlling viral infections. HIV-infected individuals develop CTL responses against epitopes derived from viral proteins, but also against cryptic epitopes encoded by viral alternative reading frames (ARF). We studied here the mechanisms of HIV-1 escape from CTLs targeting one such cryptic epitope, Q9VF, encoded by an HIV*gag* ARF and presented by HLA-B*07. Using PBMCs of HIV-infected patients, we first cloned and sequenced proviral DNA encoding for Q9VF. We identified several polymorphisms with a minority of proviruses encoding at position 5 an aspartic acid (Q9VF/5D) and a majority encoding an asparagine (Q9VF/5N). We compared the prevalence of each variant in PBMCs of HLA-B*07+ and HLA-B*07- patients. Proviruses encoding Q9VF/5D were significantly less represented in HLA-B*07+ than in HLA-B*07- patients, suggesting that Q9FV/5D encoding viruses might be under selective pressure in HLA-B*07+ individuals. We thus analyzed *ex vivo* CTL responses directed against Q9VF/5D and Q9VF/5N. Around 16% of HLA-B*07+ patients exhibited CTL responses targeting Q9VF epitopes. The frequency and the magnitude of CTL responses induced with Q9VF/5D or Q9VF/5N peptides were almost equal indicating a possible cross-reactivity of the same CTLs on the two peptides. We then dissected the cellular mechanisms involved in the presentation of Q9VF variants. As expected, cells infected with HIV strains encoding for Q9VF/5D were recognized by Q9VF/5D-specific CTLs. In contrast, Q9VF/5N-encoding strains were neither recognized by Q9VF/5N- nor by Q9VF/5D-specific CTLs. Using *in vitro* proteasomal digestions and MS/MS analysis, we demonstrate that the 5N variation introduces a strong proteasomal cleavage site within the epitope, leading to a dramatic reduction of Q9VF epitope production. Our results strongly suggest that HIV-1 escapes CTL surveillance by introducing mutations leading to HIV ARF-epitope destruction by proteasomes.

## Author Summary

In addition to the classical open reading frames encoding for the well characterized HIV proteins, HIV exhibits a vast number of alternative reading frames that have the potential to encode proteins or polypeptides. We have previously shown that such reading frames within *gag, pol* and *env* genes express T cell epitopes. In the present work, we further characterized the role of T-cell responses targeting the *gag*-overlapping reading frame in the selection of HIV variants *in vivo*. We demonstrate that under CD8+ T cell immune pressure, HIV escapes by introducing mutation that affects T-cell recognition of HIV-infected cells. We characterized the mechanism of CTL-escape and demonstrate that HIV manipulates antigen processing and presentation. Our results highlight the importance of CTL targeting these alternative reading frame-encoded antigens in the control of HIV replication.

## Introduction

Multiple lines of evidence suggest that CD8+ cytotoxic T lymphocytes (CTLs) play a critical role in controlling HIV-1 replication. During acute infection, expansion of HIV-specific CD8+ T cells (HS-CTL), before appearance of neutralizing antibodies, is associated with decreased viremia [1] and most likely determines the viral set point during chronic infection [2], [3]. Resistance to disease progression correlates with the detection of Gag-specific CTLs and with the presence of particular HLA alleles, such as HLA-B*57 and –B*27 [4], [5]. HIV rapidly mutates to evade virus-specific CD8+ T lymphocyte responses, underlying the selection pressure exerted by CTLs [2], [6], [7]–[11]. In large part due to its error prone reverse transcriptase activity, HIV possesses a unique capacity to mutate and evade CTL responses. During acute and chronic HIV infection, CTL escape mutations have been well documented [9], [12], [13]. In most cases, these mutations are intra-epitopic and affect HLA binding and/or alter TCR interactions leading to loss of CTL activation or more subtle effects [14]. However, interference with antigen processing may also lead to a reduced generation of precursor peptides and consequently peptide/MHC-I complex formation and T cell activation. This could occur at any stage of the processing pathway. Mutations in epitope-flanking regions might affect proteasomal processing or N-terminal trimming leading to escape from CTL recognition [15]–[20].

CTLs recognize peptides originating from proteasomal processing of viral proteins or truncated misfolded viral polypeptides, also called DRiPS (for defective ribosomal products) [21]–[23]. These viral polypeptides are classically derived from the fifteen HIV-1 viral proteins encoded by the nine primary open reading frames [24]. However CTLs also target peptides translated from alternative reading frames or ARFs (also called cryptic epitopes). ARF-derived peptides (ARFPs) result from a differential usage of the three-letter codon alphabet during protein synthesis. How this change of reading frame occurs remains elusive but various mechanisms have been proposed. Ribosomes can initiate translation at an internal initiation codon (Met or Cys), change reading frame by shifting, or translate alternatively spliced mRNA. Nonetheless, ARF polypeptides are processed in cells and thus constitute an important source of cryptic epitopes for MHC-I presentation [25]. CTL responses directed against these cryptic epitopes have been detected in autoimmune disease [26], in tumors [27], [28] but also in several infectious diseases, including influenza virus [29], murine AIDS [30], SIV [31] and importantly HIV infections [32]–[35].

We previously described six ARFPs presented by HLA-B*0702 overlapping the alternative reading frames of HIV-1 *gag*, *pol* or *env* genes [32]. CTL responses specific for these ARF-derived peptides were detected in the blood of HIV+ patients. In addition, HIV-infected cells were recognized by CTLs specific for the *gag*-overlapping ARF epitope (so called Q9VF/5D epitope). Importantly, we showed that the introduction of a stop codon within *gag*-ARF abrogated Q9VF/5D epitope generation and Q9VF/5D–specific CTL activation [32]. Recent studies further highlighted the *in vivo* relevance of ARFP-specific CTL responses [33], [34], [36]. In two independent cohorts studies, Bansal *et al.* and Berger *et al.* investigated the association between specific HLA alleles and HIV sequence polymorphisms within ARFs. This “HLA class I footprint approach” allowed the prediction of numerous ARFPs within the HIV-1 genome, both from sense and antisense transcripts. On a restricted number of ARFPs, they also demonstrated that these cryptic epitopes induced CTL responses during natural infection that might contribute to viral control *in vivo* [33], [34].

In the present work, we bring to light a novel mechanism of CTL escape altering the processing and presentation of the Q9VF epitope encoded by the *gag*-overlapping ARF. In PBMCs of HLA-B*07+ and HLA-B*07- HIV-infected individuals, we first compared the prevalence of QPRSNTHVF (Q9VF/5N) and QPRSDTHVF (Q9VF/5D) variants of the *gag*-ARFP. To this end, we PCR amplified and sequenced twenty HIV proviral genomes per individuals. We noticed that the proportion of proviruses encoding Q9VF/5D was significantly lower in HLA-B*07+ than in HLA-B*07- patients, suggesting that Q9FV/5D encoding viruses might be under selective pressure in HLA-B*07+ individuals. In HLA-B*07+ and HLA-B*07- patients, we analyzed *ex vivo* CTL responses directed against Q9VF/5D and Q9VF/5N and we dissected the immunogenicity of Q9VF variants. We observed that cells infected with HIV-1 strains encoding Q9VF/5N were neither recognized by Q9VF/5N- nor Q9VF/5D-specific CTLs. We demonstrate that this single amino acid (AA) variation is responsible for the lack of CD8+ T cell recognition. We show that HIV can escape CTL surveillance by introducing mutations leading to epitope destruction by proteasomes.

## Results

### Analysis of Q9VF *gag* proviral sequences and Q9VF-specific CTL responses in HLA-B*07+ patients

**Figure 1:**
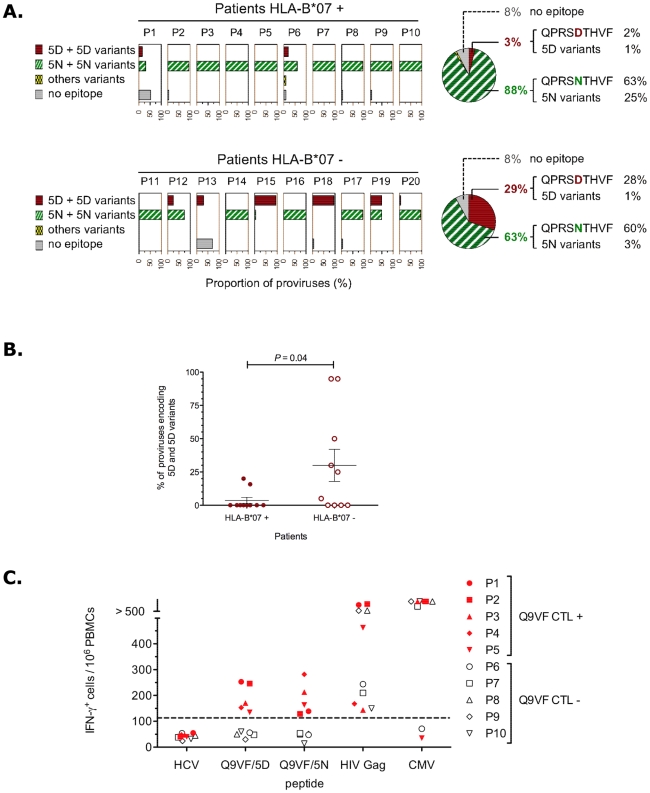
Q9VF/5D-specific CTLs exert a selection pressure on HIV Q9VF *gag*-overlapping ARF. (**A**) Analysis of Q9VF proviral sequences in HIV-infected donors. Using PBMCs, proviral DNA of 20 HIV+ individuals were extracted and the region corresponding to *gag-ARF* PCR-amplified and cloned. Twenty clones per donor were sequenced. Results are presented as percentage of provirus encoding for Q9VF/5D and 5D variants exhibiting within the epitope an additional AA difference from the consensus sequence, Q9VF/5N and 5N variants, and sequence harboring a stop codon prior the epitope (no epitope). Pies on the right represent percentage of provirus combined for all isolates. Top and bottom panels, results for HLA-B*07+ and HLA-B*07- donors, respectively. (**B**) Percentage of provirus encoding Q9VF/5D or 5D variants within HLA-B*07+ and HLA-B*07- patients. Each dot represents percentage within the PBMCs of one donor. In HLA-B*07+ patients, variants with 5D are under-represented (P<0.04). (**C**) Immunogenicity of Q9FV peptide variants. PBMCs of HIV-infected HLA-B*07+ donors were loaded with peptides and T cell activation monitored by IFNγ-ELISot. PBMCs were incubated with HLA-B*07-restricted epitopes: Q9VF/5D, Q9VF/5N, a pool of 3 immunodominant HIV-1 Gag epitopes (SPRTLNAWV, TPQDLNTML, YPLASLRSLF), a CMV-derived epitope (pp65 TPRVTGGGAM) or an HCV-derived epitope as negative control (GPRLGVRAT). Out of 31 HLA-B*07+ patients 5 reacted to Q9VF/5D and Q9VF/5N. Results for the 5 Q9VF reacting patients (Q9VF CTL +, full symbols) and 5 representative Q9VF non-reacting patients (Q9VF CTL-, open symbols) are shown. Data are means of triplicates. Dotted line indicates threshold of significant positive responses.

**Figure 2:**
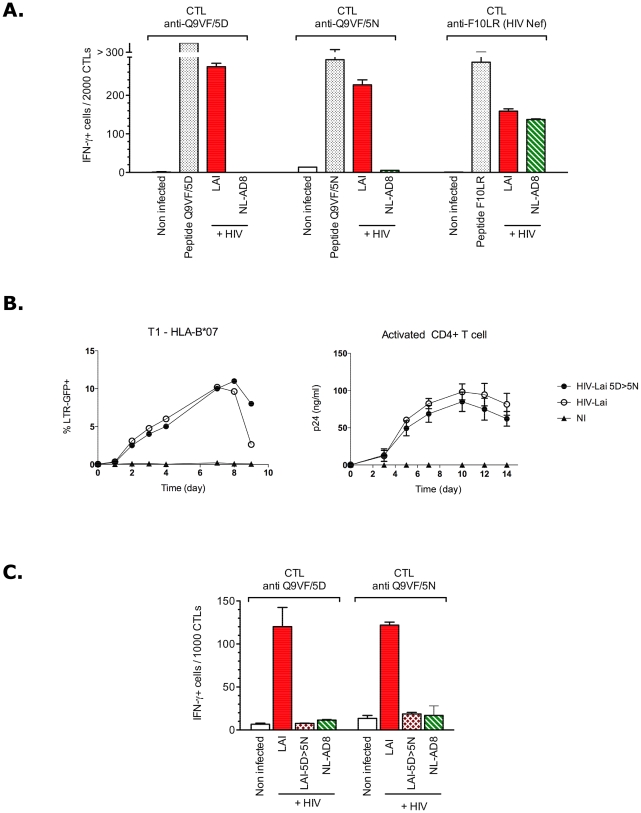
Q9VF/5D to Q9VF/5N substitution abrogates CTL recognition of HIV-infected cells. (**A**) T1-B7 cells were infected with HIV_LAI_ and HIV_NL-AD8_ expressing Q9VF/5D and Q9VF/5N, respectively. Two days p.i., the percentage of HIV-infected cells was monitored by intracellular p24 staining and flow cytometry: 50 and 47% of the cells were infected with HIV_LAI_ and HIV_NL-AD8_, respectively. In an IFNγ-ELISpot assay, infected cells were then used to activate CTL lines specific for Q9VF/5D, Q9VF/5N or an HLA-B*07-restricted HIV-1 Nef epitope (FPVTPQVPLR, F10LR) used as control. For each peptide, specific CTL lines were generated in three different HLA-B*0702 transgenic mice and used in two independent experiments. One representative experiment with one CTL line is shown (mean values of triplicates ±SD). T1-B7 cells loaded with the cognate peptide were used as positive controls. (**B**) 5N substitution does not affect HIV replication. T1-B7 cells (left panel) and CD4+ activated T cells (right panel) were infected (at 100 and 1 ng/ml respectively) with HIV_LAI_ and HIV_LAI-5D>5N_. HIV_LAI-5D>5N_ expressing Q9VF/5N was engineered by PCR mutagenesis of the HIV_LAI_ strain. Whatever the viral input (1, 10 or 100 ng/ml), 5N substitution did not alter the replication capacity of HIV_LAI-5D>5N_. T1-B7 cell infection (left panel) was monitored using GFP expression (upon trans-activation of LTR-GFP). Data are representative of at least five independent experiments using various viral inputs. CD4+ T cells infection was monitored using p24-Elisa (right panel) and correspond to the mean values (±SD) of two infections using activated CD4+ T cells from two donors and are representative of two independent experiments (using various viral input). NI: not infected. (**C**) 5N substitution is sufficient to abrogate CTL recognition of HIV-infected cells. As in (A) using T1-B7 cells infected with HIV_LAI_, HIV_NL-AD8_ and HIV_LAI-5D>5N_. Infection rates were around 30% of p24+ cells.

Q9VF was originally predicted from the sequence of the consensus HIV_HxB2_ (HIV_LAI_) isolate [32]. HIV_LAI_ bears an asparagine (N) to aspartic acid (D) substitution at position 5 (Q9VF/5D) representing less than 5% of HIV-1 clade B strains retrieved from Genbank. We decided to extend these observations by sequencing HIV proviral sequences isolated from 10 HLA-B*07+ and 10 HLA-B*07- patients. HLA-typing, virological and clinical characteristics of these patients are presented in Table 1. Both groups were age-matched and did not present any significant differences in terms of CD4 counts, viral loads or treatments (not shown). From the PBMCs of each patient, we cloned and sequenced at least 20 HIV-proviral sequences encompassing the *gag-*ARF DNA region (Figure 1A and Supplementary Figure S1). The isolated HIV sequences encoded either Q9VF/5N (present in 16 out of 20 patients, representing 62% of all isolates), Q9VF/5N variants (exhibiting within the epitope an additional AA difference from the consensus sequence, 9 out of 20 patients, 14% of all isolates) or Q9VF/5D (7 out of 20 patients, 15% of all isolates) and Q9VF/5D variants (2 out of 20 patients, 1% of all isolates) (Table 2). Between Q9VF/5N and Q9VF/5N-variants, Q9VF/5N was the major variant representing 80% of proviral sequences in this group. Q9VF/5D was the major sequence representing 94% of proviral sequences among Q9VF/5D and Q9VF/5D-variants. Note that these mutations did not impact the translation of classical *gag* ORF (Supplementary Figure S1 and not shown). In contrast, HIV proviruses harboring a STOP codon prior to Q9VF (8% of all isolates) that most likely abolishes Q9VF translation were also identified (Figure 1A). HIV proviral sequences encoding Q9VF/5N and Q9VF/5N-variants were predominant in both HLA-B*07+ and HLA-B*07- patients. Q9VF/5D or Q9VF/5D-variant HIV proviral sequences could be retrieved in two out of the ten HLA-B*07+ patients and in six out of the ten HLA-B*07- donors. Taking into consideration the diversity of HIV sequences per donor with regard to their HLA-B7 status, we observe a significant lower proportion of Q9VF/5D+ HIV strains in HLA-B*07+ than in HLA-B*07- donors (p<0.04, mean value 3% vs 29% of proviral sequences in HLA-B*07+ and HLA-B*07- donors, respectively, Figure 2B). Altogether, these results suggested that Q9VF/5D-encoding HIV strains might be under negative selective pressure in HLA-B*07+ donors. We thus analyzed CTL responses directed against Q9VF/5D and Q9VF/5N epitopes in PBMCs of patients including the 10 HLA-B*07+ patients used for the analysis of HIV proviral sequences.

**Table 1:** List of patients used in this study.

			HLA class I										
Patient	Age	Gender	A		B		C		CD4 count (cells/mL)	Time since HIV infection (yr)	Viral load^a^	Antiretroviral therapy^b^	Duration of ART (yr)
Patients HLA-B*07+													
P1	42	M	nd	nd	B*07	nd	nd	nd	491	4	<20	d4T-ddi-NVP	4
P2	33	M	nd	nd	B*07	nd	nd	nd	642	9	1776	3TC-d4T-NVP	5
P3	38	M	A*01	A*02	B*07	B*08	C*07	C*07	667	19	<20	TDF/FTC-ATV/r	17
P4	44	M	A*02	A*03	B*07	B*27	C*02	C*07	1546	22	<20	TDF/FTC-DRV/r	22
P5	46	F	A*02	A*03	B*07	B*51	C*05	C*07	414	19	<20	TDF/FTC-DRV/r-ETR-RAL	17
P6	58	M	nd	nd	B*07	nd	nd	nd	866	2.5	11482	None	
P7	41	M	nd	nd	B*07	B*18	C*05	C*07	644	17	<20	ddi/3TC-ATV/r	13
P8	47	M	A*23	A*33	B*07	B*14	C*05	C*07	892	16	<20	TDF/FTC-FPV/r	11
P9	50	M	A*02	A*03	B*07	B*44	C*07	C*07	818	14	124	ABC/3TC-LPV/r-ETR	13
P10	48	M	A*01	A*03	B*07	B*08	C*07	C*07	434	24	<20	TDF/FTC-ETR-RAL	20
Patients HLA-B*07 -													
P11	28	M	A*01	A*02	B*08	B*27	C*07	C*07	613	0.25	20293	None	
P12	44	M	A*29	A*31	B*44	B*67	C*12	C*16	319	21	<20	ABC/3TC-NVP	11
P13	53	M	A*01	A*02	B*14	B*51	C*05	C*15	351	23	<20	ddi/3TC-ATV/r	17
P14	43	M	A*01	A*68	B*14	B*15	C*04	C*05	358	24	<20	DRV/r	12
P15	63	M	A*29	A*74	B*44	B*56	C*01	C*16	1282	23	<20	TDF/FTC-ATV/r	18
P16	36	F	A*01	A*02	B*53	B*82	C*03	C*06	440	6	<20	ABC/3TC-ATV	6
P17	44	M	A*03	A*03	B*27	B*35	C*02	C*04	529	16	20	ABC/3TC-DRV/r-TDF	13
P18	44	M	A*03	A*11	B*14	B*27	C*01	C*05	1461	23	<20	TDF/FTC-EFV	0.25
P19	36	F	A*29	A*33	B*27	B*39	C*03	C*07	919	22	53	ABC/3TC-LPV/r	22
P20	23	F	A*24	A*29	B*18	B*55	C*03	C*12	96	16	38035	None	

a Copies of HIV-1 RNA per milliliter of plasma at the time of study.

b Treatment at the time of study: d4T, stavudine; ddi, diadanosine; TDF, Tenofovir; FTC, Emtricitabine; ATV, Atazanavir; r, ritonavir; DRV, Darunavir; ETR, Etravirine; LPV, Lopinavir; RAL, Raltegravir; 3TC, Lamivudine; ABC, Abacavir; EFV, Efavirenz; FPV, Fosamprenavir; NVP, Nevirapine; SQV, Saquinavir; AZT, Zidovudine; MVC, Maraviroc.

ART, antiretroviral therapy; nd, not determined.

**Table 2:** Frequencies of HIV-1 proviruses encoding Q9VF epitope variants in PBMCs of studied patients.

		Patients HLA-B*07+												Patients HLA-B*07-											
			Frequency of provirus (%)^b^												Frequency of provirus (%)^b^										
Provirus encoding Q9VF variant		Patients with provirus^a^	Mean^c^	P1	P2	P3	P4	P5	P6	P7	P8	P9	P10	Patients with provirus^a^	Mean^c^	P11	P12	P13	P14	P15	P16	P17	P18	P19	P20
Q9VF/5D+5D variants																									
5D	QPRSDTHVF	1/10	2	0	0	0	0	0	20	0	0	0	0	6/10	28	0	25	30	0	95	0	0	80	50	5
5D4G	---G-----	1/10	1	16	0	0	0	0	0	0	0	0	0	0/10	0	0	0	0	0	0	0	0	0	0	0
5D9C	--------C	0/10	0	0	0	0	0	0	0	0	0	0	0	1/10	1	0	0	0	0	0	0	0	15	0	0
Q9VF/5N+5N variants																									
5N	QPRSNTHVF	8/10	63	10	90	95	0	95	50	100	0	95	95	8/10	60	100	75	0	100	5	100	85	0	50	80
5N4G	---GN----	3/10	3	21	5	0	0	0	0	0	0	0	5	0/10	0	0	0	0	0	0	0	0	0	0	0
5N3G	--G-N----	1/10	<1	0	0	5	0	0	0	0	0	0	0	1/10	<1	0	0	0	0	0	0	0	0	0	5
5N3S	--S-N----	0/10	0	0	0	0	0	0	0	0	0	0	0	1/10	<1	0	0	0	0	0	0	5	0	0	0
5N2S	-S--N----	1/10	10	0	0	0	0	0	0	0	95	0	0	0/10	0	0	0	0	0	0	0	0	0	0	0
5N1R	R---N----	1/10	1	0	0	0	0	0	10	0	0	0	0	0/10	0	0	0	0	0	0	0	0	0	0	0
5N7Y	----N-Y--	0/10	0	0	0	0	0	0	0	0	0	0	0	1/10	<1	0	0	0	0	0	0	0	0	0	5
5N8G	----N--G-	0/10	0	0	0	0	0	0	0	0	0	0	0	1/10	<1	0	0	0	0	0	0	0	0	0	5
5N9S	----N---S	1/10	<1	0	0	0	0	5	0	0	0	0	0	0/10	0	0	0	0	0	0	0	0	0	0	0
5N9L	----N---L	0/10	0	0	0	0	0	0	0	0	0	0	0	1/10	<1	0	0	0	0	0	0	5	0	0	0
5N9C	----N---C	1/10	10	0	0	0	100	0	0	0	0	0	0	0/10	0	0	0	0	0	0	0	0	0	0	0
Other variants																									
5Y	QPRSYTHVF	1/10	1	0	0	0	0	0	10	0	0	0	0	0/10	0	0	0	0	0	0	0	0	0	0	0
No epitope		5/10	8	53	5	0	0	0	10	0	5	5	0	3/10	8	0	0	70	0	0	0	5	5	0	0

a Number of patients in which at least one proviral clone encodes the Q9VF variant epitope/total number of tested patients.

b Frequency of proviral clones encoding Q9VF variant epitope among the twenty clones sequenced per patient.

c Average frequency of proviruses among the ten studied patients (HLA-B*07+ or HLA-B*07-).

PBMCs from 31 HLA-B*07+ patients were loaded with various peptides and submitted to IFNγ-ELISpot (Figure 1C and not shown). Incubations with peptides corresponding to well-characterized HLA-B*0702-restricted immunodominant epitopes from HIV-1 Gag classical ORF (SPRTLNAWV, TPQDLNTML, YPLASLRSLF) induced a significant IFNγ-release, demonstrating that in the course of natural infection the donors mounted CTL responses to HIV-1 antigens. Five out of the 31 HLA-B*07+ donors showed a low but significant activation with Q9VF/5D and Q9VF/5N peptides (Figure 1C). Note that donors reacted to both peptides or reacted to none and that the frequencies of CTL responding to Q9VF/5D and Q9VF/5N peptides were in the same order of magnitude (from 150 to 300 CTL per million of PBMCs), suggesting that the reactivity to one or the other peptide might be due to cross reactivity. We previously demonstrated that CTL lines raised against Q9VF/5N were indeed cross-reactive on Q9VF/5D and *vice versa* ([32] and Supplementary Figure S2).

Viruses encoding Q9VF/5D were not isolated from PBMCs of the five Q9VF responders (Figure 1), with the exception of patients P1 that harbored proviruses encoding a Q9VF/5D variant (QPRG**D**THVF, representing 16% of sequences in this donor). These data prompt us to study the immunogenicity of the Q9VF/5N and Q9VF/5D epitope variants.

### Q9VF/5D to 5N substitution abrogates CTL recognition of HIV-infected cells

We asked whether the Q9VF/5N epitope was processed and presented to HS-CTLs by HIV-infected cells. HLA-B*0702+ cells were infected with HIV_LAI_ and HIV_NL-AD8_ strains encoding Q9VF/5D or Q9VF/5N respectively. Five days post-infection (pi), 50 and 47% of the cells were productively infected by HIV_LAI_ and HIV_NL-AD8_ respectively (as monitored by intracellular Gag-p24 FACS-staining (not shown)). Infected cells were then co-cultured with HIV-specific CTL lines and T cell activation measured using IFNγ-ELISpot assays (Figure 2). HLA-transgenic mice offer a rapid and convenient model to identify human T cell epitopes [24] and to generate CTL lines specific for peptides of unknown immunogenicity in humans, such as Q9VF/5N. For this reason, Q9VF/5D- and Q9VF/5N-specific CTL lines were generated by peptide immunization of HLA-B*0702+ transgenic mice and *in vitro* restimulations [32], [37]. As expected, Q9VF/5D- and Q9VF/5N- specific CTLs secreted high levels of IFNγ in response to Q9VF/5D and Q9VF/5N peptide loaded cells respectively (Figure 2A). Note that Q9VF/5D- and Q9VF/5N-specific CTL lines displayed similar capacity to recognize peptide-loaded cells (Supplementary Figure S2), suggesting that the Q9VF/5N variant affects neither MHC nor TCR binding of the peptide. As we previously reported [32], HIV_LAI_-infected cells induced a robust activation of Q9VF/5D-specific CTLs. Due to their capacity to cross-react on Q9VF/5D peptide (Supplementary Figure S2 and [32]), Q9VF/5N-specific CTLs were also stimulated by HIV_LAI_-infected cells, thus demonstrating that these CTL lines are fully competent in recognizing HIV-infected cells. In contrast, Q9VF/5D- and Q9VF/5N-specific CTLs were not activated upon co-culture with HIV_NL-AD8_-infected cells (Figure 2A). This is not due to the incapacity of HIV_NL-AD8_-infected cells to activate HS-CTLs since CTL clones specific for an HLA-B*0702-restricted HIV-1 Nef epitope (F10LR), raised as a control in these experiments, were activated upon co-culture with HIV_LAI_- and HIV_NL-AD8_-infected cells.

To extend these observations to other HIV-1 isolates, HLA-B*0702+ cells were also infected with HIV_MN_ that encodes for Q9VF/5N and used as target cells to activate Q9VF/5D- and Q9VF/5N-specific CTLs (Supplementary Figure S3). HIV_NL-AD8_- and HIV_MN_-infected cells did not induce Q9VF/5D- nor Q9VF/5N-specific CTL activation. Overall, these results suggested that HIV-infected cells did not present the Q9VF/5N peptide.

Epitope flanking regions have a direct impact on antigen processing and presentation [38]. Thereafter, to exclude the possibility that HIV sequence variations outside the Q9VF/5N peptide might be responsible for the lack of presentation, we introduced in HIV_LAI_ a D to N mutation within the Q9VF epitope (so called HIV_LAI-5D>5N_). This mutation did not affect the primary open reading frame of Gag (Supplementary Figure S1) and did not alter viral replication in T cell lines or primary CD4+ T cells (Figure 2B). However, cells infected with HIV_LAI-5D>5N_ could not activate Q9VF/5D- nor Q9VF/5N-specific CTLs (Figure 2C). Thereafter, this single amino acid substitution was sufficient to abrogate CTL recognition, thus indicating that this asparagine alters Q9VF MHC-I presentation. We then sought to dissect the mechanism responsible for the lack of Q9VF/5N MHC-I presentation.

### Q9VF/5N binds TAP pumps and HLA-B*0702 molecules

**Figure 3:**
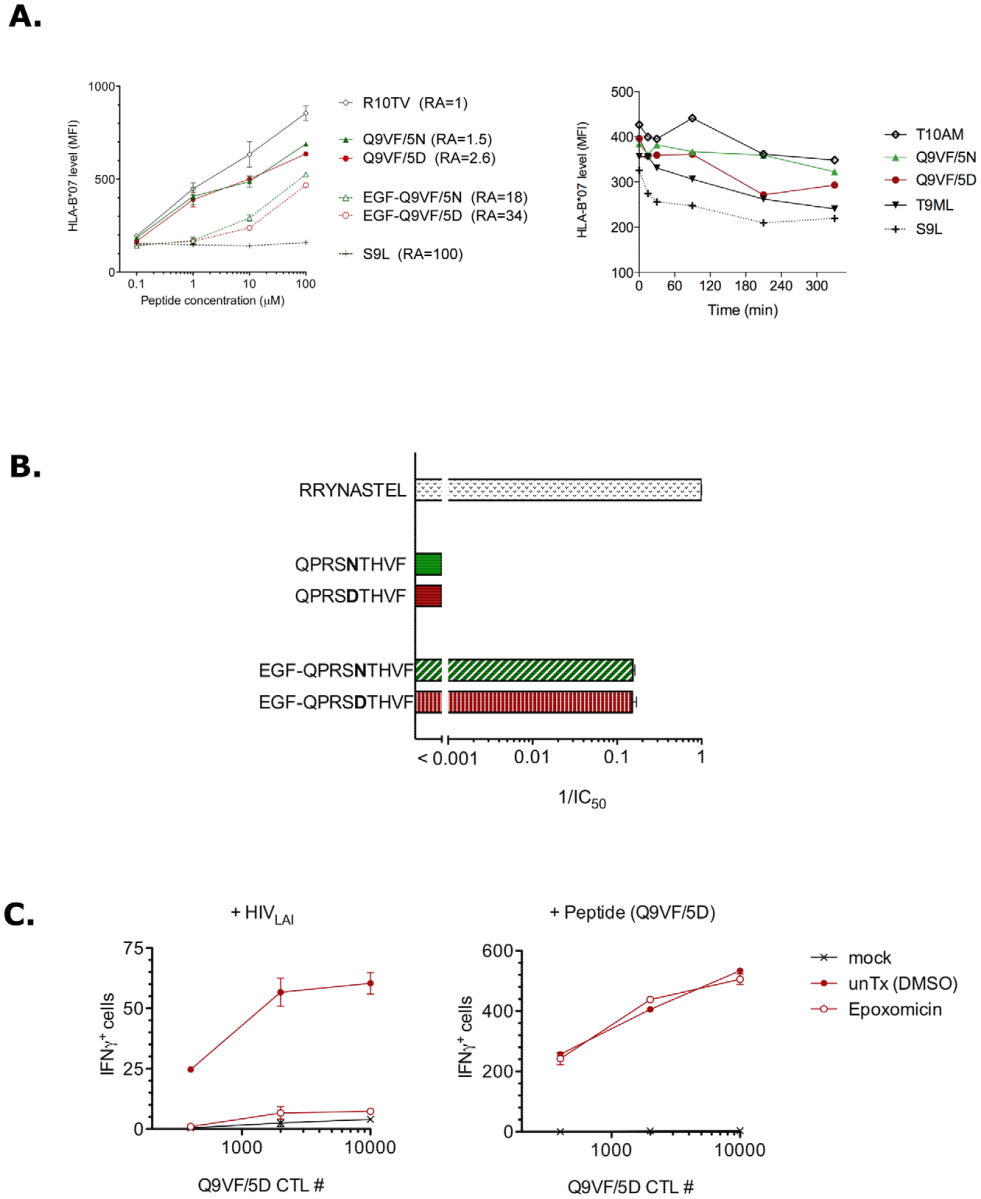
Q9VF/5N binds TAP pumps and HLA-B*0702 molecules. (**A**) Q9VF/5N and Q9VF/5D peptides exhibit similar affinities for HLA-B*0702. (*Left panel*) Q9VF/5D, Q9VF/5N and their natural EGF Nt-extended precursors were loaded O/N at RT on T2-B7 cells. An HLA-B*07-restricted CMV-derived reference epitope (pp65 RPHERNGFTV, R10TV) and an HLA-A*02-restricted HIV-1-derived epitope (p17 SLYNTVATL, SL9) were also used as positive and negative control, respectively. HLA-B*0702 binding was monitored using ME-1 antibody and flow cytometry. Based on the reference peptide R10TV, a relative affinity (RA) was calculated. Data are representative of three different experiments (mean values of triplicates ±SD). (*Right panel*) T2-B7 were cultured O/N at 26°C to increase peptide-receptive cell surface molecules, pulsed with the indicated peptides for 2 h in presence of β2-microglobulin and BFA to stop delivery of newly synthesized MHC-I molecules. Cells were then shifted to 37°C for 1 h, washed to remove unbound peptides and incubated at 37°C in presence of BFA (0.5 µg/ml) which is considered as time “zero”. At the indicated time points, samples were removed to 0°C, stained on ice using ME.1 Ab and analyzed by FACS. Data are mean values of two independent experiments. The capacity of each peptide to stabilize HLA-B*0702 (*t*_1/2_) was compared using exponential regression. T_1/2_ of HLA-B*0702 pulsed with the irrelevant peptide (S9L) was 22 min while binding of Q9VF/5D and Q9VF/5N peptides prolonged the t_1/2_ to 211 and 641 min respectively. T_1/2_ of CMV (pp65 TPRVTGGGAM, T10AM) and Gag (p24 TPQDLNTML, T9ML) peptides used as positive were 552 and 124 min respectively. (**B**) Human TAP transporter binding assay. Microsomes from insect cells expressing human TAPs were incubated with the labeled reference reporter peptide (RRYNASTEL, R9L) then loaded with serial dilutions of unlabeled reference peptide or tested peptides with or without EGF Nt-extension. TAP affinities were determined as the concentrations required to inhibit 50% of reporter peptide binding (IC_50_) and data are presented as 1/IC_50_ ratios: the highest the ratio, the stronger the affinity. Results are mean values (±SD) from three independent experiments. (**C**) Q9VF/5D epitope generation is dependent on proteasomal processing. T1-B7 cells were infected with HIV_LAI_ (as in Figure 1), monitored for HIV infection by flow cytometry, treated or not (unTx) with epoxomicin (6 h at 37°C). To remove residual MHC-peptide complexes, cells were then treated with a citrate-phosphate buffer, washed and used as targets to activate Q9VF/5D-specific CTLs in IFNγ-ELISpot assay (8h). Note that epoxomycin inhibition affected neither MHC-density (as monitored by FACS, not shown) nor the capacity of treated cells to present exogenous peptide (0.1 µg/ml) (right panel). Results are mean values (±SD) of triplicates and representative of three different Q9VF/5D CTL clones. Mock, non infected cells (left panel) or loaded with the irrelevant HCV peptide (right panel).

The capacity of antigenic peptides to bind to a given HLA allele is determined by the so-called anchor residues [39]. Mutating an anchor residue abrogates peptide HLA-binding and subsequent T cell activation, a strategy often used by viruses to escape viral-specific T cell responses. The anchor residues of HLA-B*0702 reside at position 2 and 9 of the peptide-ligands. Thereafter, the D to N substitution at position 5 was not predicted to influence Q9VF peptide binding to HLA-B*0702 [40]. However, besides anchor residues, auxiliary residues might affect peptide binding, we thus compared the capacity of Q9VF/5D and Q9VF/5N peptides to bind HLA-B*0702. To this end, T2-HLA-B*0702 cells were loaded O/N with Q9VF/5D or Q9VF/5N peptides and binding to HLA-B*0702 molecules at the cell surface monitored by FACS (Figure 3A, left panel). Q9VF/5D and Q9VF/5N peptides exhibited similar capacities to bind HLA-B*0702 with a relative affinity (RA, based on the reference peptide) of 2.6 and 1.5 respectively (Figure 3A, left panel). To further characterize the impact of the 5D to 5N substitution on peptide-MHC interactions, we compared the capacity of the peptides to stabilize HLA-B*0702 molecules at the cell surface of T2-HLA-B*0702 (Figure 3A, right panel). To this end, T2-HLA-B*0702 were cultured O/N at 26°C to allow surface expression of peptide-receptive MHC molecules, loaded with a high concentration of peptides, shifted to 37°C and the stability of HLA-B*0702-peptide complexes monitored by FACS at various time points. An exponential regression of HLA-B*0702 mean fluorescence intensity (MFI) vs. time reveals that the stability (t_1/2_) of HLA-B*0702 pulsed with an irrelevant peptide (S9L) is 22 min while binding of Q9VF/5D and Q9VF/5N peptides prolongs the t_1/2_ to 211 and 641 min respectively (Figure 3A, right panel). Thereafter, Q9VF/5D and Q9VF/5N peptides are very good HLA-B*0702-binders and 5D to 5N substitution tends to prolong surface expression of HLA-B*0702.

Precursor peptides are transported by the TAP pumps (transporter associated with antigen processing) from the cytosol into the endoplasmic reticulum (ER), and then loaded on nascent MHC-I molecules [41]. N-terminally extended peptide precursors are also transported and further trimmed in the ER by the endoplasmic reticulum aminopeptidase ERAAP and bound to MHC-I molecules [42], [43]. We asked whether the absence of Q9VF/5N peptide presentation by HLA-B*0702 within infected cells might be the result of inefficient ER-translocation of the Q9VF/5N epitope and/or Q9VF/5N-peptide precursors by TAP. Hence, we used a TAP-binding assay [44] to evaluate the affinities of Q9VF/5D and Q9VF/5N and their precursors with TAP. Q9VF/5D and Q9VF/5N exhibited a poor affinity for TAP (Figure 3B), most likely due to the presence of a proline at position 2 that negatively impacts on TAP-mediated peptide transport [44]. In contrast, their N-terminally extended peptide precursors EGF-Q9VF/5D and EGF-Q9VF/5N showed at least a two-log increased efficiency to compete for TAP with an equal 1/IC_50_ of 0.15. Whatever the precursor, Q9VF/5D and Q9VF/5N containing peptides did not show differences in their capacity to bind human TAP molecules.

Overall, these data demonstrated that the D to N substitution within Q9VF does not impact on TAP transport and HLA binding. In contrast, the 5N substitution might prolong epitope presentation on the cell surface.

### Q9VF/5D epitope generation is dependent on proteasomal cleavages

The proteasomes, that are the major catalytic enzymes involved in antigen processing, generate the carboxyl termini of most MHC-bound peptides [38], [45]. We thus asked whether the generation of Q9VF/5D was dependent on proteasomal processing. To this end, HLA-B*0702+ cells were infected with HIV_LAI_. Five days pi, infected cells were incubated with a potent and selective proteasome inhibitor, epoxomicin [46], treated with a citrate-phosphate buffer to remove residual MHC-peptide complexes, washed and cultured with Q9VF/5D-specific CTLs as previously described. Epoxomicin treatment abolished the capacity of HIV_LAI_-infected cells to activate Q9VF/5D-specific CTLs, as measured in IFNγ-ELISpot (Figure 3C, left panel). Note that epoxomycin inhibition affected neither MHC-density (as monitored by FACS, not shown) nor the capacity of treated cells to present exogenous peptide (at 0.1 µg/ml) (Figure 3C, right panel). Thereafter, these results demonstrated that the generation of Q9VF epitope depends on proteasomal processing.

### 5N introduces an aberrant proteasomal cleavage site within Q9VF epitope

Proteasomes might also destroy CTL epitopes by generating aberrant cleavages within the epitope [47] or in epitope-flanking regions [19], [48]. We thus asked whether aberrant proteasomal cleavages might be responsible for the lack of Q9VF/5N presentation.

**Figure 4:**
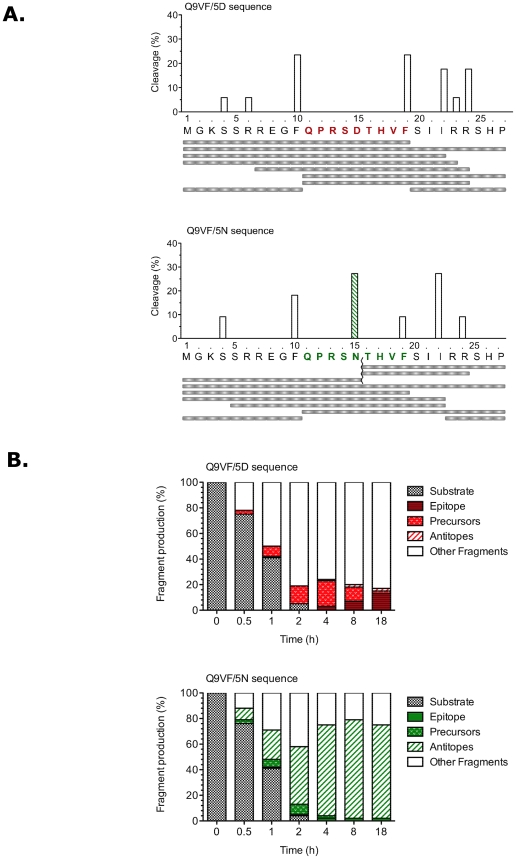
5N introduces an aberrant proteasomal cleavage site within the epitope. (**A**) 5N introduces a strong cleavage site within Q9VF epitope. 27mer synthetic peptides encompassing Q9VF/5D or Q9VF/5N were submitted to *in vitro* immunoproteasome (IP) digestion. Resulting peptide fragments were analyzed by mass-spectrometry. Proteasome cleavage patterns are presented as C-terminal cleavages to a specific AA (horizontal axis) of Q9VF/5D (upper panel) and Q9VF/5N (lower panel) substrates. The percentage of C-terminal cuts at each AA is indicated. The most frequent fragments at 1 h IP digestion are depicted. Data represent one of two independent experiments. (**B**) The overall production of Q9VF epitope is drastically reduced by the 5N substitution. Q9VF/5D (upper panel) and Q9VF/5N (lower panel) encompassing peptides were digested by IP from 0 h to 18 h. Resulting peptide fragments were analyzed by MS/MS, as in (A). Proteasome cleavage patterns are presented as the estimated percentage of peptide fragments corresponding to either the substrate (M1-P27), the epitope Q9VF (Q11-F19), precursors with a C-terminal cut at F19, peptide fragments with a cleavage within the epitope most likely abolishing epitope production (referred to as “Antitopes”), or other fragments, with the sum of all fragments intensities set as 100%.

The proteasome is a large multicatalytic protease composed of standard and inducible subunits that replace the standard subunits upon exposure to IFNγ and form the so-called “immunoproteasomes” (IP). IP is found in most cell types after IFNγ-exposure, but is constitutive in APCs and is induced in HIV-infected T cells [49]. Standard (SP) and IP proteasomes display discrete differences in their capacity to cleave a given peptide substrate [50]. We submitted the full-length polypeptides from the *gag*-overlapping ARF to IP processing. 27mer peptides encompassing Q9VF/5D or Q9VF/5N peptides were synthesized and incubated with IP purified from T2.27 cells [51]. After 1 h incubation, the digestions were analyzed by mass spectrometry (RP-HPLC SI) and peptide fragments identified by MS/MS (Figure 4A). IP digestion of Q9VF/5D encompassing peptide showed the presence of major proteasomal cleavage sites after amino acids F10, F19, I22 and R24 representing around 80% of total cleavages. The cleavage at position F19 generated the C-terminal cut of the N-extended precursors of Q9VF (M1-F19). After 1 h incubation, when comparing the IP digestion profiles of Q9VF/5D and Q9VF/5N encompassing peptides, we noticed the presence of a new cleavage site within the Q9VF/5N epitope. This cut at position N15 was the most prevalent among Q9VF/5N representing up to 28% of total IP cleavages. These results demonstrated that the D to N substitution introduces a major cleavage site within the Q9VF/5N epitope. Nonetheless the C-terminal cut necessary for the generation of Nt-extended Q9VF/5N precursors was also detected following 1 h of proteasomal digestion.

Thereafter, we sought to evaluate the amount of cleavage products generated during Q9VF/5D and Q9VF/5N digestions. To this end, we performed kinetics of IP digestion where aliquots were regularly collected and submitted to mass spectrometry analysis as before (Figure 4B). To compare the amounts of cleavage products, we used the MS fragment intensity as a surrogate marker for quantity since these two parameters correlate significantly [15]. The variations among the different fragments generated are presented as the relative intensity of peptides that exhibit a Q9VF C-terminal cut (epitope or precursors) or peptides issued from cleavages within the Q9VF epitope (referred to as the antitopes) (Figure 4B). Kinetics of digestion of peptides encompassing either Q9VF/5D or Q9VF/5N were identical: 24%, 59% and 96% of both substrates was degraded after 30 min, 1 h and 2 h respectively. At latter time points, both 27mers were undetectable. In the course of Q9VF/5D substrate digestion, the precursor (M1-F19) was readily produced starting from 30 min with a peak at 4 h digestion (representing 20% of digested products). The epitope was detected starting from 1 h digestion and accumulated reaching 13% of all peptide fragments at time 18 h. At latter time points, Q9VF/5D epitopes and precursors represented up to 14% of all peptide fragments detected. An antitope corresponding to a cleavage at position S14 was also generated but represented less than 2% of detected fragments at each time point. In contrast, during Q9VF/5N substrate digestion, the antitopes corresponding to the cleavage at position N15 were already produced after 30 min of digestion and reached around 77% of all peptides from 4 to 18 h, further demonstrating that N15 is a major cleavage site within Q9VF/5N. Interestingly, during Q9VF/5N digestion, the epitope was barely detected even at latter time points (less than 2% of digested products). The precursor M1-F19 accumulated from 30 min to 2 h (8% of digested products) but was undetectable after 4 h, suggesting that the cleavage at position N15 destroyed this peptide. Overall, the amounts of Q9VF/5N epitope and precursors produced were markedly reduced as compared to Q9VF/5D digestion.

Taken together, these results demonstrate that the Q9VF/5D epitope is efficiently produced by proteasomes and accumulates with time. In contrast, the D to N substitution introduces a major cleavage site within the epitope leading to the destruction of the Q9VF/5N epitope and thus the absence of MHC-I binding and presentation.

## Discussion

The three-letter codon alphabet allows protein synthesis in six possible overlapping reading frames. A vast number of ARFs have the potential to encode proteins or epitopic peptides (ARFPs). Using an “HLA class I footprint” approach, Bansal *et al* and Berger *et al* recently predicted the existence of numerous ARFPs within HIV-1 genome [33], [34]. We have previously shown that ARFP-specific CTLs are induced during natural infection [32]. These CTL responses might contribute to viral control driving HIV evolution at the population level. ARFPs can mutate during the first year of infection, suggesting a possible selection of escapes variants [33], [34]. Such a scenario has been highlighted in the macaque model of SIV infection [31]. Mamu-B*17+ macaques generate strong CTL responses against SIV ARF-encoded epitopes leading to ARF mutation affecting epitope binding to Mamu-B*17 molecules and subsequent SIV replication rebound [31]. In the present study, we characterized a novel mechanism of ARFP-specific CTL escape resulting from HIV epitope destruction by the proteasomes. We suggest that ARFP-specific CTLs exert a selection pressure leading to negative selection of targeted HIV strains. Overall, our work shows that CTL escape mutations are not limited to epitopes encoded by classical ORF, highlighting the role of ARFP-specific CTLs in the control of HIV infection.

We previously identified a panel of epitopes encoded by ARFs within HIV-1 *gag*, *pol* and *env* genes [32]. The *gag*-overlapping ARF encoding for the Q9VF epitope presented by HLA-B*0702 drew our attention due to its polymorphism. In a cross-sectional cohort study, we report that proviruses encoding the Q9VF/5D epitope (and 5D variants) are rare and significantly under-represented in PBMCs of HLA-B*07+ patients, thus suggesting Q9VF/5D-specific CTLs might exert a negative selection pressure on HIV strains encoding Q9VF/5D variants. In HIV-1 *gag* ARF, the virus might escape CTL immune pressure by introducing a 5D to 5N substitution or Stop codons but prior the epitope. We thus analyzed CTL responses directed against Q9VF/5D and Q9VF/5N epitopes in PBMCs of patients. Q9VF/5D and Q9VF/5N peptides induced CTL responses in 16% of HLA-B*07+ individuals tested. Donors reacted to both peptides or reacted to none. The frequencies of CTLs responding to Q9VF/5D and Q9VF/5N peptides were about the same magnitude, suggesting that the reactivity to one or the other peptide might be due to cross reactivity. The frequency and magnitude of Q9VF/5D responses in HLA-B*07+ patients were rather low as compared to immunodominant HLA-B*07-restricted responses (Figure 1 and [24]). This might be due to the fact that the patients included in the study were under retroviral therapy that might affect the expression of ARF during residual HIV-1 translation (Table 1). Alternatively in our assays, we are most likely monitoring memory responses to Q9VF/5D that are usually of low magnitude. This possibility is supported by the observation from Bansal *et al* that ARFP encoding sequences mutate during the first year of infection [33]. Overall, the low representation of Q9VF/5D encoding HIV proviral sequences in PBMCs of HLA-B*07+ individuals and the low frequency and magnitude of CTL responses to Q9VF/5D strongly supported our initial hypothesis that 5N substitution is an escape mutation.

We dissected the immunogenicity of the Q9VF/5N epitope. We showed that cells infected with HIV-1 strains encoding Q9VF/5N (HIV_NL-AD8_ and HIV_MN_) were not recognized by Q9VF/5N-specific CTLs. In contrast, Q9VF/5N- and Q9VF/5D-specific CTLs were activated by HIV-1 strains encoding Q9VF/5D (HIV_LAI_). We demonstrated that the single AA substitution from 5D to 5N in HIV_LAI_ sequence is sufficient and required to abrogate CTL recognition of HIV-infected cells. Thereafter, the acquisition of this 5N mutation by HIV might help the virus to interfere with Q9VF epitope expression or processing and presentation.

Viruses can interfere with antigen expression to escape CTL lysis [23]. Various mechanisms have been proposed for the biosynthesis of ARF-derived polypeptides. Ribosomes can scan through conventional initiation codons [29], initiate translation at an internal initiation non-AUG-codons (Leu or Cys) [34], [52], change reading frame by shifting [53], or translate alternatively spliced mRNA (for review see [25]). We previously described the presence of a conserved slippery motif (UUUAAAU) upstream of *gag*-ARF start codon that may facilitate ribosomal slippage and thus Q9VF synthesis [32]. Interestingly, a structured region (hairpin) in HIV-1 RNA has been identified downstream of this slippery motif [53]. This highly structured RNA region might cause ribosomal pausing during *gag* translation thus facilitating ribosomal slippery and Q9VF expression. The D to N substitution within the Q9VF epitope is translated from a codon that is located in the flexible loop of the RNA hairpin structure [53]. Although it remains to be formally proven, this D to N substitution most likely does not impact the RNA structure and hence Q9VF expression.

Viruses also manipulate antigen processing and presentation to escape CTL responses. Interference with antigen presentation could arise at any stage in the pathway, including processing by proteasomes, binding of epitope-precursors to TAP, destruction of these precursors by peptidases in the ER or cytosol and peptide binding to the MHC-I molecule. HIV-specific CTL responses have been shown repeatedly to select for intra-epitope mutations that affect HLA-binding or TcR recognition. In addition, HIV escape mutations outside the epitope (extra-epitope mutations) can interfere with antigen processing by proteasomes [17]–[19], [47], [54], [55] or by the ER aminopeptidase ERAAP [16]. To our knowledge, intra-epitope mutations affecting antigen processing have not been described thus far. Several studies proposed that intra-epitope variation might affect processing but did not provide a mechanism [34], [20]. The only evidence that intra-epitope mutations might affect proteasomal processing of viral antigens comes from mouse models [47], [56].

We provide several lines of evidence strongly suggesting that the D to N substitution within the Q9VF epitope impacts neither TcR recognition nor MHC binding: i) Q9VF/5N- and Q9VF/5D-specific CTLs can be generate upon peptide immunization of HLA-B*07-transgenic mice and cross-react to the alternate peptide ([32] and Supplementary Figure S2); and ii) Q9VF/5N and Q9VF/5D peptides bind HLA-B*0702 (Figure 3A). In addition, we show that Q9VF/5N and Q9VF/5D peptide and their precursors (elongated on the N-termini) efficiently bind TAP, thus demonstrating that the D to N substitution does not affect peptide translocation into the ER. As previously observed with peptides bearing a proline at position 2 [44], the optimal Q9VF/5N- and Q9VF/5D epitopes had a reduced capacity to bind TAP as compared to their Nt-extended precursors (Figure 3B), suggesting that in the ER peptide-trimming is required for proper HLA-B*0702 binding. The ER aminopeptidase ERAAP provides peptides for many MHC-I molecules but has been also implicated in the destruction of CTL epitopes [16]. However, ERAAP cannot process X-P motifs in peptide sequences [42]. Thereafter, though it cannot be formally excluded, a role of ERAAP in the destruction of Q9VF/5N is very unlikely. Overall, these data support the concept that the intra-epitope D to N substitution interferes with proteasomal processing. Using *in vitro* proteasomal digestions, we demonstrate that the D to N substitution introduces a major cleavage site within the Q9VF epitope (at position N15). Note that at 1 h-digestion time point we identify mainly primary cleavage products since less than 50% of the peptide substrates (the 27mer) have been digested (Figure 4A). To further highlight the potential impact of this N15 cleavage site in the generation of the Q9VF epitope, we performed kinetics of peptide digestion using IP. We observed that amounts of Q9VF/5N epitope and precursors produced were markedly reduced as compared to Q9VF/5D. These results strongly suggest that proteasome cleavages at position N15 destroy the Q9VF/5N epitope and precursors resulting in the lack of MHC-I presentation and CTL activation. In conclusion, a single amino acid variation within HIV epitope can result in epitope destruction and absence of HIV-specific CTL activation.

Mutation in HIV-1 genome can be silent or can differentially impact the fitness of the virus. Due to the redundancy of the codon alphabet, the 5D to 5N substitution in Q9VF does not impact the primary *gag*-ORF and thus viral replication (Figure 2B). Nevertheless, considering the multitude of existing ARFs, some mutations within ARF encoding sequences most likely affect viral fitness and these ARF sequences might be unavoidably conserved throughout HIV-1 isolates. Thereafter, the great diversity of ARF epitopes produced during HIV infection offers a vast panel of therapeutic targets to stimulate CTL responses. It is interesting to note that ARF-specific CD8+ T cells can performed multiple functions [33], [34] and control viral replication *in vitro*, characteristics that correlate with slow disease progression [57]. In addition, CTLs targeting ARF-derived epitopes can be induced upon vaccination [58] and tumor infiltrating CTLs specific for ARFPs have been also identified in various cancers, including melanoma and breast cancers [25]. Such responses against crytptic epitopes represent a great potential for future immunotherapeutic strategies.

## Materials and Methods

### Study population

HIV-1-infected peripheral blood mononuclear cells (PBMCs) were obtained from HCV (Hepatitis C virus) negative French ALT-ANRS-CO15 cohort patients [59]. The 31 HLA-B*07+ and 10 HLA-B*07- individuals were identified using the anti-HLA-B*07 antibody ME.1. HLA status was further confirmed by genotyping using PCR [60] or using the Luminex xMAP technology [61]. HLA-typing, virological and clinical characteristics of the ten HLA-B*07+ and ten HLA-B*07- patients included in the study are presented in Table 1.

### Ethics statement

Patient samples were collected according to French Ethical rules. Written informed consent and approval by institutional review Board at the Pitié-Salpêtrière Hospital were obtained.

Animals were bred at the Pasteur Institute. The Office of Laboratory Animal Care at Pasteur Institute reviewed and approved protocols for compliance with the French and European regulations on Animal Welfare and with Public Health Service recommendations (Directive 2010/63/EU).

### Human CTL assays

PBMCs were isolated by ficoll-centrifugation, pulsed with Q9VF peptides (1 µM, 1 h at 37°C), and submitted to IFNγ-ELISpot assays as previously described [46]. The HLA-B*0702-restricted peptides used were: HCV-derived epitope G9AT (GPRLGVRAT), CMV-derived epitope T10AM (pp65 _417_TPRVTGGGAM_426_) used as negative and positive control respectively and a pool of known Gag HIV-1-derived epitopes (p24 _16_SPRTLNAWV_24_, p24 _48_TPQDLNTML_56_, p2p7p1p6 _121_YPLASLRSLF_130_) as control for HIV reactivity [24]. Responses were considered positive when IFNγ production was superior to 50 spots/10^6^ PBMCs and at least threefold higher than background (measured with the HCV peptide).

### Mouse CTL recognition of infected T1 cells

Mouse CTL lines were derived from splenocytes of peptide immunized HLA-B*07^mα3^ transgenic mice. In brief, these mice express HLA-B*0702 heavy chain with a murine α3 domain and their H-2K^b^ and H-2D^b^ class Ia genes have been inactivated [37]. Cytolytic activity of splenocyte cultures was first assessed in a^51^Cr release assay [32]. Peptide specific CTL lines were stimulated *in vitro* (5 µg/mL of peptide) and cultured in RPMI 1640 medium supplemented with 10% FCS, 0.5 µM 2-β-mercaptoethanol (Sigma), 100 IU/mL penicillin and 100 µg/mL streptomycin (Gibco-BRL). Ten days later, 2×10^3^, 400 and 80 CTLs in triplicates were stimulated by 10^5^ HIV-1-infected T1-B7 cells and IFNγ release was detected by ELISpot assay. Cross-reactivity of Q9VF/5D- and Q9VF/5N-specific CTLs was tested in IFNγ-ELISpot and Cr^51^-release assays [32] using T1-B7 peptide-loaded cells. Mouse CTL lines specific for the HLA-B*0702-restricted HIV-1 Nef-derived epitope F10LR (Nef _68_FPVTPQVPLR_77_; [22]) were used as controls. When stated, HIV-infected T1-B7 cells were treated with epoxomicin (6 h, 1 µg/ml, Calbiochem). To remove residual MHC-peptide complexes, epoxomycin-exposed cells were treated with a citrate-phosphate buffer (pH 3.3) containing 1% BSA and washed twice, prior co-culture with CTLs for an additional 8 h.

### Virus and infections

HIV_LAI 5D>5N_ was generated by a single amino acid mutation in HIV_LAI_ provirus. The GAT codon (D) of Gag-ARF (AA in position 15) was replaced by an AAT codon (N) without affecting the primary Gag AA coding sequence, using the following primer (5′-GGC TTT CAG CCC AGA AGT AAT ACC CAT GTT TTC AGC) and Quickchange XL Site-directed Mutagenesis Kit (Stratagene). HIV_LAI_, HIV_LAI-5D>5N_, HIV_NL-AD8_ and HIV_MN_ were produced by transfection of 293T cells using routine procedures [62]. T1 cells (174xCEM, CCR5+ LTR-GFP+) stably transfected with the HLA-B v T1-B7 cells, [53]) were infected and used as antigen-presenting cells. 5×10^6^ T1-B7 cells were infected with 500 ng of p24 for 3 h in culture medium containing 10 mM Hepes and 4 µg/ml DEAE-dextran. 2 to 5 days p.i., infected T1-B7 cells were used as antigen-presenting cells in IFNγ-ELISpot assay. For the infection kinetics, T1-B7 cells were infected with the indicated viruses according to the same procedure using 1, 10 or 100 ng/ml of p24. Primary CD4+ T cells were isolated from the blood of healthy donors using ficoll centrifugation and magnetic beads (Miltenyi) and activated using PHA (1 µg/ml, PAA) and rhIL-2 (50 IU/ml, Chiron) [62]. Seven days post activation, CD4+ PHA blasts were infected with various doses of HIV (from 1 to 100 ng/ml of p24). HIV infection was monitored by FACS (Becton Dickinson) using intracellular HIV p24 staining (KC57 Ab, Beckman Coulter) or p24-Elisa (PerkinElmer).

### Sequencing of the Gag-ARF encoding region from clonal HIV-1 populations

Total DNA was extracted from PBMCs of HLA-B*07+ and HLA-B*07- HIV+ patients using QIAampblood DNA minikit (Qiagen). To analyze the diversity of HIV-1 proviruses in the PBMCs of patients, a 267-bp fragment encompassing the Gag-ARF coding sequence was amplified by nested PCRs as followed: 5 min of initial denaturation at 94°C, 1 min at 94°C, 1 min at 57°C, and 1 min at 72°C for 30 cycles, followed by 7 min at 72°C. The outer primer pair used was (5′- ATC AAG CTT GCA CAG CAA GCA GCA GCT GAC) and (5′- CAG GAA CTA CTA GTA CCC TTC AGG AAT TCG G), and the inner primer pair was (5′- TAC CCT ATA GTG CAG AAC ATC CAG GG) and (5′- GAT AGA GTG CAT CCA GTG CAT GCA). Samples were treated separately and negative controls were systematically included. Purified PCR products were cloned using a TOPO-TA cloning kit (Invitrogen). Twenty clones per patient were isolated and *gag-*ARF inserts from each clonal DNA plasmid were amplified by PCR using M13 primers and sequenced (Applied Biosystem).

### HLA-B*07.02-peptide binding and stabilization assays

The capacity of the peptides to bind HLA-B*0702 was determined using a classical HLA stabilization assays with the TAP-deficient cell line T2 HLA-B*0702+ [37]. Briefly, cells were incubated overnight with 100, 10, 1 and 0.1 µM of peptide in serum-free medium at room temperature. Cells were then stained with the anti-HLA-B*07 ME.1 antibody and HLA-B*07 surface expression analyzed by FACS (Becton Dickinson). The concentration needed to reach 50% of the maximal fluorescence (as defined with the R10TV peptide (CMV pp65 _265_RPHERNGFTV_274_) was calculated (IC_50_). The relative affinity (RA) is the IC_50_ ratio of the tested and R10TV reference peptide (the lower the relative affinity, the stronger the binding). The HLA-A*02-restricted peptide S9L (HIV-1 p17 _77_SLYNTVATL_85_) was used as negative control. To monitor the capacity of the peptides to stabilize HLA-B*0702, T2-HLA-B*0702 were cultured O/N at 26°C and pulsed the last 2 h with peptide (100 µM) in presence of β2-microglubilin (Sigma, 1 µg/ml) and brefeldin-A (BFA, Sigma, 10 µg/ml). Cells were then shifted to 37°C for 1 h, washed to remove unbound peptides and incubated at 37°C in presence of BFA (0.5 µg/ml). Samples were removed to 0°C at the indicated time points. Cells were then stained at 4°C using the ME.1 antibody and analyzed by FACS. Data (HLA-B*0702 expression) are expressed as MFI vs. time. The capacity of each peptide to stabilize HLA-B*07 (*t*_1/2_)is deduced from an exponential regression (one phase decay) using Prism software. A constrain corresponding to the MFI value obtained for the irrelevant peptide (S9L) at the latest time point was applied to the plateaus. T10AM (pp65 _417_TPRVTGGGAM_426_) and T9ML (p24 _48_TPQDLNTML_56_) peptides were used as positive controls.

### TAP-binding assay

The capacity of the peptides to bind TAP was measured in a competitive binding assay as described previously [44]. Briefly, microsomes were purified from Sf9 insect cells expressing human TAP1–TAP2 complexes, pulsed with the iodinated reporter peptide R9L (RRYNASTEL) at 300 nM, and loaded with a dilution of competitor test peptides (0.1 to 1,000 fold molar excess relative to radioactive reporter peptide). TAP affinities were determined as the concentrations required to inhibit 50% of reporter peptide binding (IC_50_). Results are expressed as 1/IC_50_ ratios and are mean values from three independent experiments. The highest the 1/IC_50_ ratio, the highest the affinity.

### *In vitro* proteasome digestions

Immunoproteasomes were isolated from T2.27mp cells (that stably express all three immunosubunits) as previously described [51]. Purified proteasomes were analyzed by SDS-PAGE. The yield was calculated at 90–95%. The 27mer peptides encompassing Q9VF/5D or Q9VF/5N were synthesized using standard Fmoc method on an Applied Biosystems 433A automated synthesizer. The peptides were purified by HPLC and analyzed by mass spectrometry. Three nmol of peptides were digested *in vitro* using 1 µg of proteasomes (for 0.5, 1, 2, 4, 8 and 18 h) in 100 µl of buffer containing 20 mM Hepes/KOH, pH 7.8, 2 mM magnesium acetate and 2 mM dithiothreitol. Reactions were stopped by the addition of trifluoroacetic acid to a final concentration of 0.3%. The digestions were analyzed, by mass spectrometry (RP-HPLC ESI) and the products were identified by MS/MS.

### Statistical analysis

A standard two-tailed nonparametric Mann-Whitney *U-*test (with P<0.05 considered significant) was used to perform statistical comparison of HIV-1 proviral sequences frequencies using statistical analysis Prism software (GraphPad).

## Supporting Information

Figure S1 Amino acid and nucleotide sequences of Gag and Gag-ARF. (**A**) Nucleotide and corresponding amino acid sequences of Gag (frame 1) and Gag-ARF (frame 3, bold) are depicted. Nucleotide numbering is according to HIV_HXB2_ sequence. ATG start and TGA stop codons of *Gag-ARF* are in bold and the Q9VF/5D epitope is underlined. (**B**) Nucleotide and amino acid sequences of Gag and Gag-ARF from HIV_LAI_, HIV_NL-AD8_, HIV_MN_ and HIV_LAI-5D>5N_ strains.
(TIF)

Figure S2 Q9VF/5D and Q9VF/5N CTL cross-reactivity. The cross-reactivity of Q9VF/5D- and Q9VF/5N-specific CTLs (generated in HLA-B*0702 transgenic mice) was tested in IFNγ-ELISpot (**A**) and Cr^51^-release assays (**B**) using T1-B7 cells loaded with a single dose (1 µg/ml) (**A**) or a titration (**B**) of Q9VF/5D or Q9VF/5N peptides. A CMV-derived HLA-B*07-restricted epitope (RPHERNGFTV, R10TV) was used as negative control. Q9VF/5D- and Q9VF/5N-specific CTLs displayed similar capacity to recognize cells loaded with their cognate peptides. CTLs were also equally activated by the alternate peptides. Data are mean values of triplicates (±SD) and representative of at least three independent experiments.
(TIF)

Figure S3 Q9VF/5N encoding HIV strains are not recognized by Q9VF-specific CTLs. As in Figure 2A using T1-B7 cells infected with HIV_LAI_, HIV_NL-AD8_ or HIV_MN_ (X4-tropic isolate encoding Q9VF/5N). Infection rates were equivalent (around 30% of p24+ cells). Infected cells were then used in an IFNγ-ELISpot assay to activate Q9VF/5D- and Q9VF/5N-specific CTLs. For each peptide, specific CTL lines were generated in three different HLA-B*0702 transgenic mice and used in two independent experiments. One representative experiment with one CTL line is shown (mean values of triplicates±SD).
(TIF)

## References

[1] Wang YE, Li B, Carlson JM, Streeck H, Gladden AD (2009). Protective HLA class I alleles that restrict acute-phase
CD8+ T-cell responses are associated with viral escape mutations
located in highly conserved regions of human immunodeficiency virus type
1.. J Virol.

[2] Goonetilleke N, Liu MK, Salazar-Gonzalez JF, Ferrari G, Giorgi E (2009). The first T cell response to transmitted/founder virus
contributes to the control of acute viremia in HIV-1
infection.. J Exp Med.

[3] Koup RA, Safrit JT, Cao Y, Andrews CA, Leod GM (1994). Temporal association of cellular immune responses with the
initial control of viremia in primary human immunodeficiency virus type 1
syndrome.. J Virol.

[4] Kiepiela P, Leslie AJ, Honeyborne I, Ramduth D, Thobakgale C (2004). Dominant influence of HLA-B in mediating the potential
co-evolution of HIV and HLA.. Nature.

[5] Kiepiela P, Ngumbela K, Thobakgale C, Ramduth D, Honeyborne I (2007). CD8+ T-cell responses to different HIV proteins have
discordant associations with viral load.. Nat Med.

[6] Brumme ZL, John M, Carlson JM, Brumme CJ, Chan D (2009). HLA-associated immune escape pathways in HIV-1 subtype B Gag, Pol
and Nef proteins.. PLoS One.

[7] Duda A, Lee-Turner L, Fox J, Robinson N, Dustan S (2009). HLA-associated clinical progression correlates with epitope
reversion rates in early human immunodeficiency virus
infection.. J Virol.

[8] Goulder PJ, Brander C, Tang Y, Tremblay C, Colbert RA (2001). Evolution and transmission of stable CTL escape mutations in HIV
infection.. Nature.

[9] Kawashima Y, Pfafferott K, Frater J, Matthews P, Payne R (2009). Adaptation of HIV-1 to human leukocyte antigen class
I.. Nature.

[10] Koizumi H, Hashimoto M, Fujiwara M, Murakoshi H, Chikata T (2010). Different in vivo effects of HIV-1 immunodominant
epitope-specific CTLs on selection of escape mutant viruses.. J Virol.

[11] Philips RE, Rowland-Jones S, Nixon DF, Gotch FM, Edwards JP (1991). Human immunodeficiency virus genetic variation that can escape
cytotoxic T cell recognition.. Nature.

[12] Borrow P, Lewicki H, Wei X, Horwitz MS, Peffer N (1997). Antiviral pressure exerted by HIV-1-specific cytotoxic T
lymphocytes (CTLs) during primary infection demonstrated by rapid selection
of CTL escape virus.. Nat Med.

[13] Goulder PJ, Phillips RE, Colbert RA, McAdam S, Ogg G (1997). Late escape from an immunodominant cytotoxic T-lymphocyte
response associated with progression to AIDS.. Nat Med.

[14] Goulder PJ, Watkins DI (2004). HIV and SIV CTL escape: implications for vaccine
design.. Nat Rev Immunol.

[15] Tenzer S, Wee E, Burgevin A, Stewart-Jones G, Friis L (2009). Antigen processing influences HIV-specific cytotoxic T lymphocyte
immunodominance.. Nat Immunol.

[16] Draenert R, Le Gall S, Pfafferott KJ, Leslie AJ, Chetty P (2004). Immune selection for altered antigen processing leads to
cytotoxic T lymphocyte escape in chronic HIV-1 infection.. J Exp Med.

[17] Le Gall S, Stamegna P, Walker BD (2007). Portable flanking sequences modulate CTL epitope
processing.. J Clin Invest.

[18] Milicic A, Price DA, Zimbwa P, Booth BL, Brown HL (2005). CD8+ T cell epitope-flanking mutations disrupt proteasomal
processing of HIV-1 Nef.. J Immunol.

[19] Zimbwa P, Milicic A, Frater J, Scriba TJ, Willis A (2007). Precise identification of a human immunodeficiency virus type 1
antigen processing mutant.. J Virol.

[20] Yokomaku Y, Miura H, Tomiyama H, Kawana-Tachikawa A, Takiguchi M (2004). Impaired processing and presentation of cytotoxic-T-lymphocyte
(CTL) epitopes are major escape mechanisms from CTL immune pressure in human
immunodeficiency virus type 1 infection.. J Virol.

[21] Schubert U, Anton LC, Gibbs J, Norbury CC, Yewdell JW (2000). Rapid degradation of a large fraction of newly synthesized
proteins by proteasomes.. Nature.

[22] Casartelli N, Guivel-Benhassine F, Bouziat R, Brandler S, Schwartz O (2010). The antiviral factor APOBEC3G improves CTL recognition of
cultured HIV-infected T cells.. J Exp Med.

[23] Cardinaud S, Starck SR, Chandra P, Shastri N (2010). The synthesis of truncated polypeptides for immune surveillance
and viral evasion.. PLoS One.

[24] Cardinaud S, Bouziat R, Rohrlich PS, Tourdot S, Weiss L (2009). Design of a HIV-1-derived HLA-B07.02-restricted polyepitope
construct.. Aids.

[25] Ho O, Green WR (2006). Alternative translational products and cryptic T cell epitopes:
expecting the unexpected.. J Immunol.

[26] Saulquin X, Scotet E, Trautmann L, Peyrat MA, Halary F (2002). +1 Frameshifting as a novel mechanism to generate a cryptic
cytotoxic T lymphocyte epitope derived from human interleukin
10.. J Exp Med.

[27] Wang RF, Parkhurst MR, Kawakami Y, Robbins PF, Rosenberg SA (1996). Utilization of an alternative open reading frame of a normal gene
in generating a novel human cancer antigen.. J Exp Med.

[28] Godet Y, Moreau-Aubry A, Guilloux Y, Vignard V, Khammari A (2008). MELOE-1 is a new antigen overexpressed in melanomas and involved
in adoptive T cell transfer efficiency.. J Exp Med.

[29] Bullock TN, Eisenlohr LC (1996). Ribosomal scanning past the primary initiation codon as a
mechanism for expression of CTL epitopes encoded in alternative reading
frames.. J Exp Med.

[30] Mayrand SM, Schwarz DA, Green WR (1998). An alternative translational reading frame encodes an
immunodominant retroviral CTL determinant expressed by an
immunodeficiency-causing retrovirus.. J Immunol.

[31] Maness NJ, Valentine LE, May GE, Reed J, Piaskowski SM (2007). AIDS virus specific CD8+ T lymphocytes against an
immunodominant cryptic epitope select for viral escape.. J Exp Med.

[32] Cardinaud S, Moris A, Fevrier M, Rohrlich PS, Weiss L (2004). Identification of cryptic MHC I-restricted epitopes encoded by
HIV-1 alternative reading frames.. J Exp Med.

[33] Bansal A, Carlson J, Yan J, Akinsiku OT, Schaefer M (2010). CD8 T cell response and evolutionary pressure to HIV-1 cryptic
epitopes derived from antisense transcription.. J Exp Med 207: 51-59,.

[34] Berger CT, Carlson JM, Brumme CJ, Hartman KL, Brumme ZL (2010). Viral adaptation to immune selection pressure by HLA class
I-restricted CTL responses targeting epitopes in HIV frameshift
sequences.. J Exp Med 207: 61-75,.

[35] Schweighardt B, Wrin T, Meiklejohn DA, Spotts G, Petropoulos CJ (2010). Immune escape mutations detected within HIV-1 epitopes associated
with viral control during treatment interruption.. J Acquir Immune Defic Syndr.

[36] Garrison KE, Champiat S, York VA, Agrawal AT, Kallas EG (2009). Transcriptional errors in human immunodeficiency virus type 1
generate targets for T-cell responses.. Clin Vaccine Immunol.

[37] Rohrlich PS, Cardinaud S, Firat H, Lamari M, Briand P (2003). HLA-B*0702 transgenic, H-2K(b)D(b) double-knockout mice:
phenotypical and functional characterization in response to influenza
virus.. Int Immunol.

[38] Kloetzel PM (2001). Antigen processing by the proteasome.. Nat Rev Mol Cell Biol.

[39] Falk K, Rotzschke O, Stevanovic S, Jung G, Rammensee HG (1991). Allele-specific motifs revealed by sequencing of self-peptides
eluted from MHC molecules.. Nature.

[40] Rammensee H, Bachmann J, Emmerich NP, Bachor OA, Stevanovic S (1999). SYFPEITHI: database for MHC ligands and peptide
motifs.. Immunogenetics.

[41] Lauvau G, Kakimi K, Niedermann G, Ostankovitch M, Yotnda P (1999). Human transporters associated with antigen processing (TAPs)
select epitope precursor peptides for processing in the endoplasmic
reticulum and presentation to T cells.. J Exp Med.

[42] Serwold T, Gaw S, Shastri N (2001). ER aminopeptidases generate a unique pool of peptides for MHC
class I molecules.. Nat Immunol.

[43] Saric T, Beninga J, Graef CI, Akopian TN, Rock KL (2001). Major histocompatibility complex class I-presented antigenic
peptides are degraded in cytosolic extracts primarily by thimet
oligopeptidase.. J Biol Chem.

[44] van Endert PM, Riganelli D, Greco G, Fleischhauer K, Sidney J (1995). The peptide-binding motif for the human transporter associated
with antigen processing.. J Exp Med.

[45] Cascio P, Hilton C, Kisselev AF, Rock KL, Goldberg AL (2001). 26S proteasomes and immunoproteasomes produce mainly N-extended
versions of an antigenic peptide.. Embo J.

[46] Moris A, Nobile C, Buseyne F, Porrot F, Abastado JP (2004). DC-SIGN promotes exogenous MHC-I-restricted HIV-1 antigen
presentation.. Blood.

[47] Ossendorp F, Eggers M, Neisig A, Ruppert T, Groettrup M (1996). A single residue exchange within a viral CTL epitope alters
proteasome-mediated degradation resulting in lack of antigen
presentation.. Immunity.

[48] Chapiro J, Claverol S, Piette F, Ma W, Stroobant V (2006). Destructive cleavage of antigenic peptides either by the
immunoproteasome or by the standard proteasome results in differential
antigen presentation.. J Immunol.

[49] Gavioli R, Gallerani E, Fortini C, Fabris M, Bottoni A (2004). HIV-1 tat protein modulates the generation of cytotoxic T cell
epitopes by modifying proteasome composition and enzymatic
activity.. J Immunol.

[50] Toes RE, Nussbaum AK, Degermann S, Schirle M, Emmerich NP (2001). Discrete cleavage motifs of constitutive and immunoproteasomes
revealed by quantitative analysis of cleavage products.. J Exp Med.

[51] Kuckelkorn U, Ferreira EA, Drung I, Liewer U, Kloetzel PM (2002). The effect of the interferon-gamma-inducible processing machinery
on the generation of a naturally tumor-associated human cytotoxic T
lymphocyte epitope within a wild-type and mutant p53 sequence
context.. Eur J Immunol.

[52] Schwab SR, Li KC, Kang C, Shastri N (2003). Constitutive display of cryptic translation products by MHC class
I molecules.. Science.

[53] Watts JM, Dang KK, Gorelick RJ, Leonard CW, Bess JW (2009). Architecture and secondary structure of an entire HIV-1 RNA
genome.. Nature.

[54] Allen TM, Altfeld M, Geer SC, Kalife ET, Moore C (2005). Selective escape from CD8+ T-cell responses represents a
major driving force of human immunodeficiency virus type 1 (HIV-1) sequence
diversity and reveals constraints on HIV-1 evolution.. J Virol.

[55] Parcej D, Tampe R (2010). ABC proteins in antigen translocation and viral
inhibition.. Nat Chem Biol.

[56] Basler M, Youhnovski N, Van Den Broek M, Przybylski M, Groettrup M (2004). Immunoproteasomes down-regulate presentation of a subdominant T
cell epitope from lymphocytic choriomeningitis virus.. J Immunol.

[57] Almeida JR, Price DA, Papagno L, Arkoub ZA, Sauce D (2007). Superior control of HIV-1 replication by CD8+ T cells is
reflected by their avidity, polyfunctionality, and clonal
turnover.. J Exp Med.

[58] Maness NJ, Wilson NA, Reed JS, Piaskowski SM, Sacha JB (2010). Robust, vaccine-induced CD8(+) T lymphocyte response against
an out-of-frame epitope.. J Immunol.

[59] Martinez V, Costagliola D, Bonduelle O, N'go N, Schnuriger A (2005). Combination of HIV-1-specific CD4 Th1 cell responses and IgG2
antibodies is the best predictor for persistence of long-term
nonprogression.. J Infect Dis.

[60] Bunce M, O'Neill CM, Barnardo MC, Krausa P, Browning MJ (1995). Phototyping: comprehensive DNA typing for HLA-A, B, C, DRB1,
DRB3, DRB4, DRB5 & DQB1 by PCR with 144 primer mixes utilizing
sequence-specific primers (PCR-SSP).. Tissue Antigens.

[61] Dunbar SA (2006). Applications of Luminex xMAP technology for rapid,
high-throughput multiplexed nucleic acid detection.. Clin Chim Acta.

[62] Moris A, Pajot A, Blanchet F, Guivel-Benhassine F, Salcedo M (2006). Dendritic cells and HIV-specific CD4+ T cells: HIV antigen
presentation, T-cell activation, and viral transfer.. Blood.

