# Supplementary figures and images for: CTL Escape Mediated by Proteasomal Destruction of an HIV-1 Cryptic Epitope
Source: PLoS Pathog. 2011 May 12;7(5):e1002049. doi: 10.1371/journal.ppat.1002049 (PMC3093368; doi:10.1371/journal.ppat.1002049)

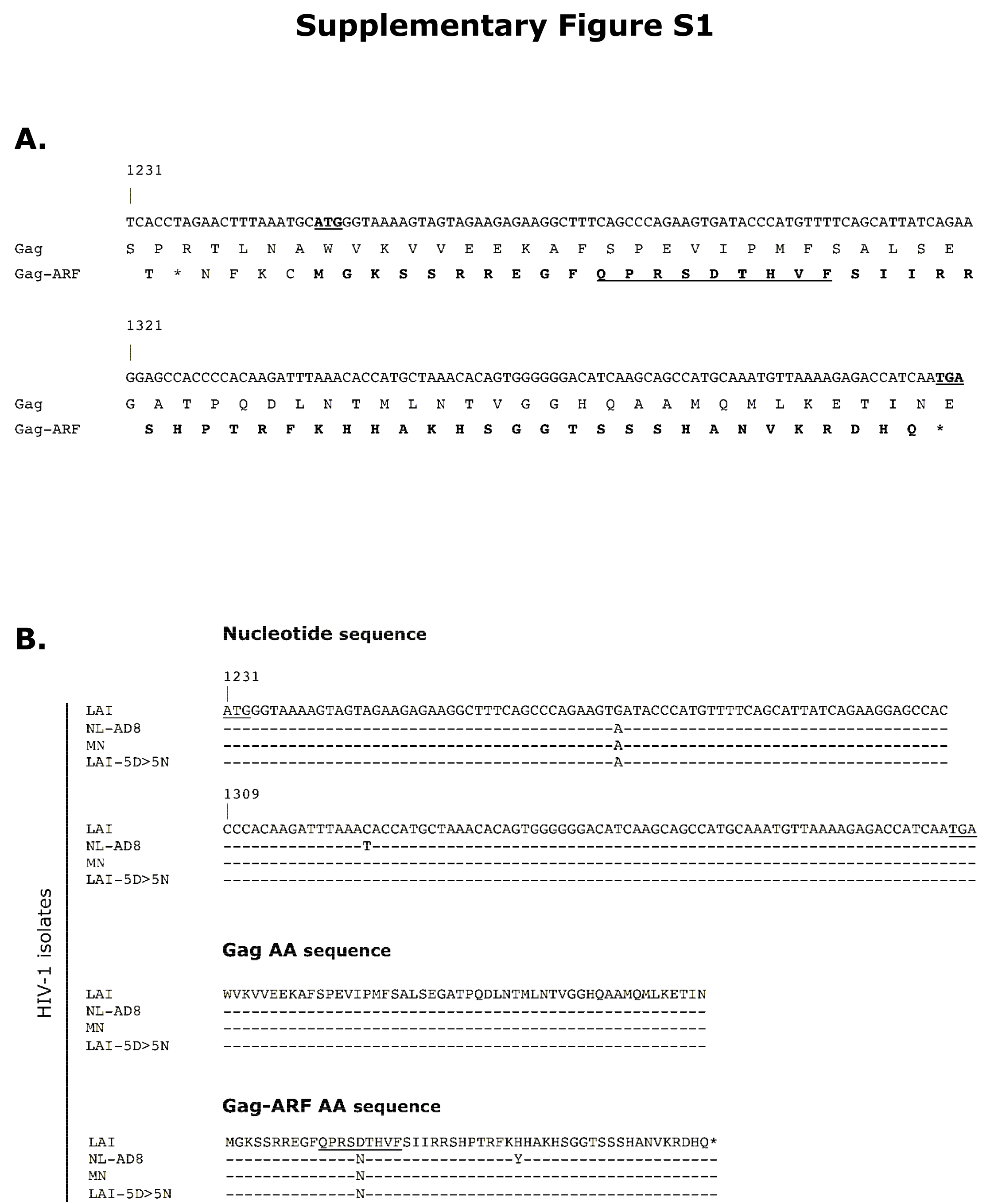

Supplement: Figure S1 — Amino acid and nucleotide sequences of Gag and Gag-ARF. (A) Nucleotide and corresponding amino acid sequences of Gag (frame 1) and Gag-ARF (frame 3, bold) are depicted. Nucleotide numbering is according to HIVHXB2 sequence. ATG start and TGA stop codons of Gag-ARF are in bold and the Q9VF/5D epitope is underlined. (B) Nucleotide and amino acid sequences of Gag and Gag-ARF from HIVLAI, HIVNL-AD8, HIVMN and HIVLAI-5D>5N strains. (TIF) [file ppat.1002049.s001.tif]

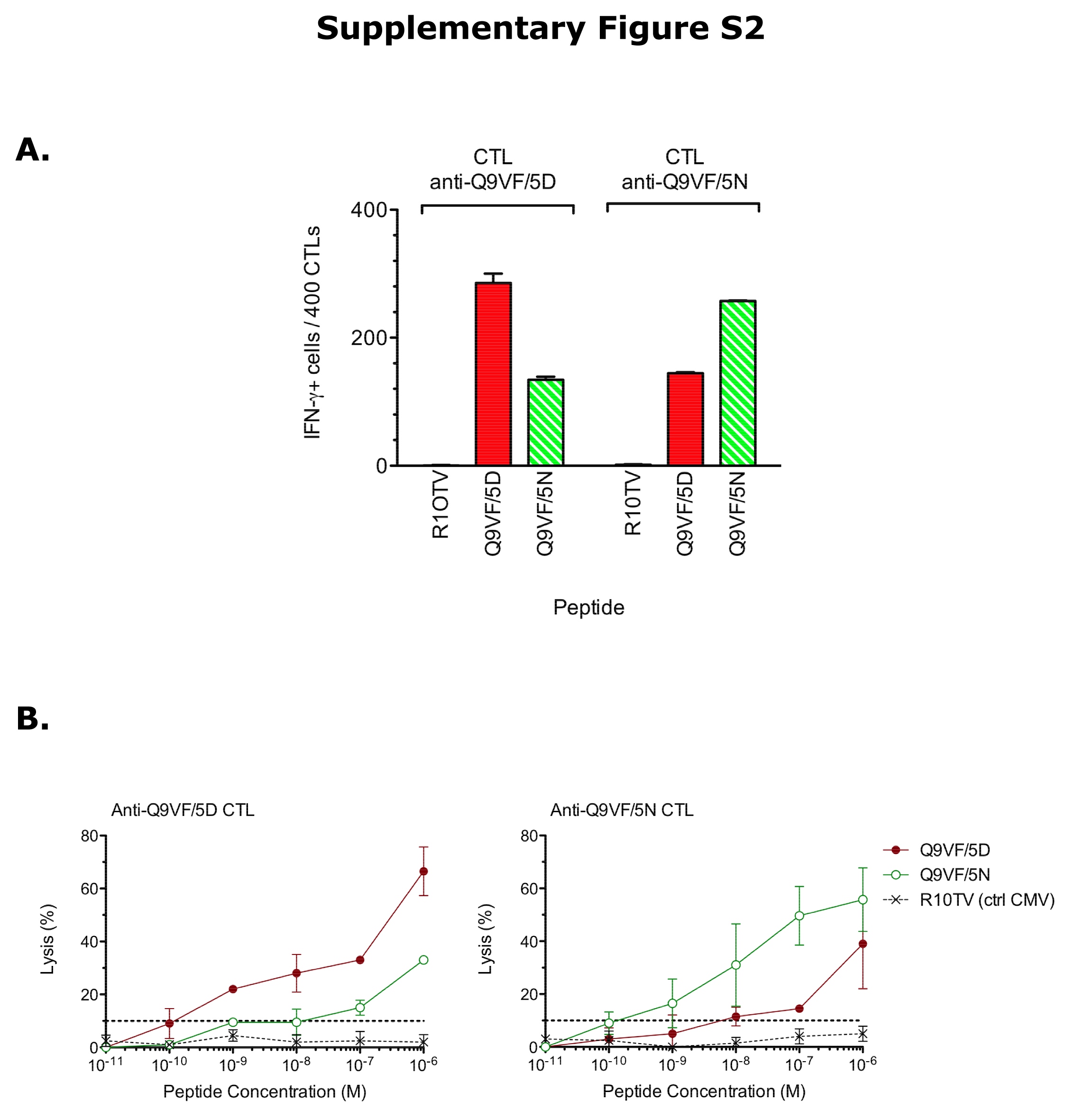

Supplement: Figure S2 — Q9VF/5D and Q9VF/5N CTL cross-reactivity. The cross-reactivity of Q9VF/5D- and Q9VF/5N-specific CTLs (generated in HLA-B*0702 transgenic mice) was tested in IFNγ-ELISpot (A) and Cr51-release assays (B) using T1-B7 cells loaded with a single dose (1 µg/ml) (A) or a titration (B) of Q9VF/5D or Q9VF/5N peptides. A CMV-derived HLA-B*07-restricted epitope (RPHERNGFTV, R10TV) was used as negative control. Q9VF/5D- and Q9VF/5N-specific CTLs displayed similar capacity to recognize cells loaded with their cognate peptides. CTLs were also equally activated by the alternate peptides. Data are mean values of triplicates (±SD) and representative of at least three independent experiments. (TIF) [file ppat.1002049.s002.tif]

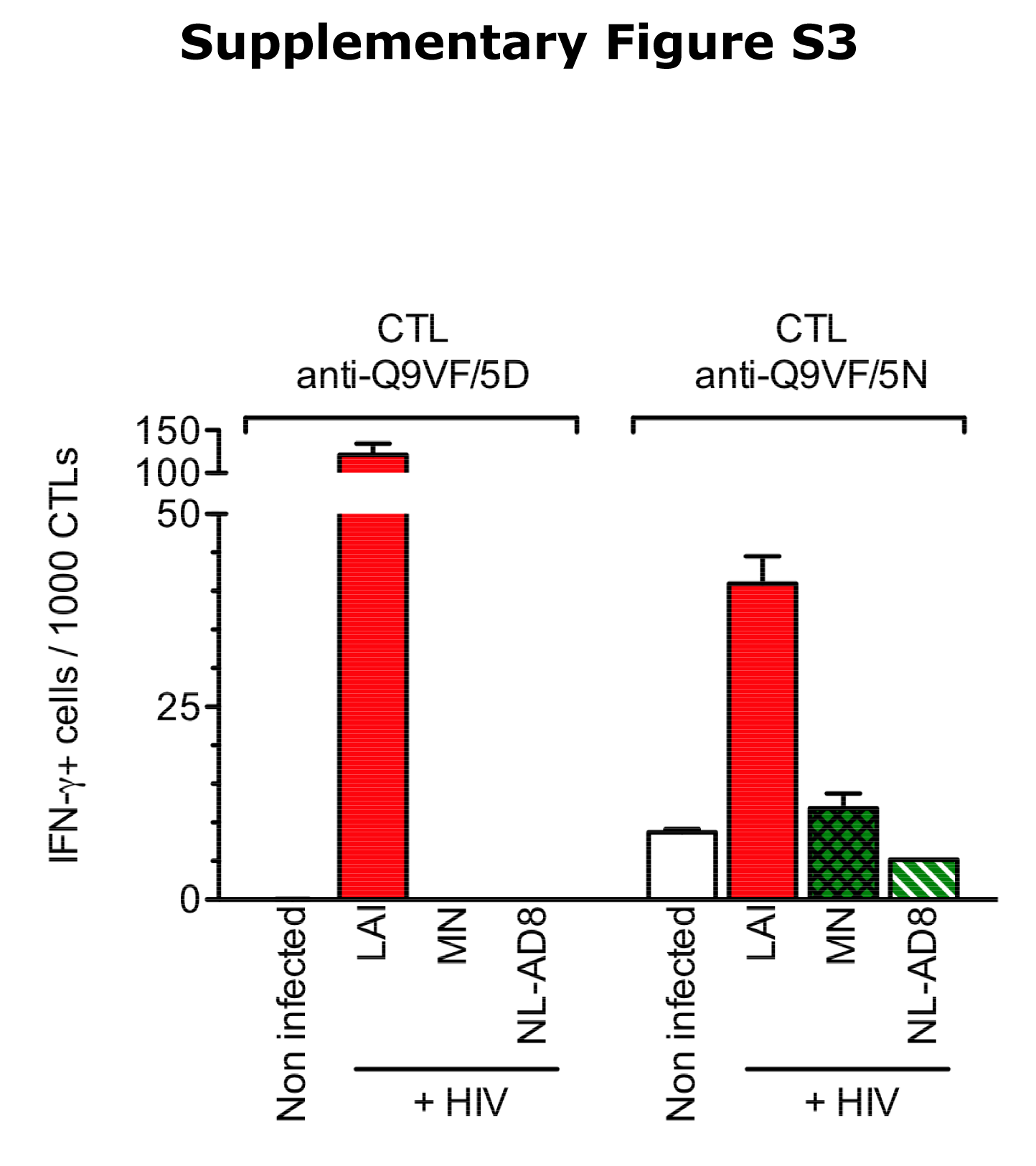

Supplement: Figure S3 — Q9VF/5N encoding HIV strains are not recognized by Q9VF-specific CTLs. As in Figure 2A using T1-B7 cells infected with HIVLAI, HIVNL-AD8 or HIVMN (X4-tropic isolate encoding Q9VF/5N). Infection rates were equivalent (around 30% of p24+ cells). Infected cells were then used in an IFNγ-ELISpot assay to activate Q9VF/5D- and Q9VF/5N-specific CTLs. For each peptide, specific CTL lines were generated in three different HLA-B*0702 transgenic mice and used in two independent experiments. One representative experiment with one CTL line is shown (mean values of triplicates±SD). (TIF) [file ppat.1002049.s003.tif]
